# Modulation of Antimicrobial Host Defense Peptide Gene Expression by Free Fatty Acids

**DOI:** 10.1371/journal.pone.0049558

**Published:** 2012-11-15

**Authors:** Lakshmi T. Sunkara, Weiyu Jiang, Guolong Zhang

**Affiliations:** 1 Department of Animal Science, Oklahoma State University, Stillwater, Oklahoma, United States of America; 2 Department of Biochemistry and Molecular Biology, Oklahoma State University, Stillwater, Oklahoma, United States of America; 3 Department of Physiological Sciences, Oklahoma State University, Stillwater, Oklahoma, United States of America; Southern Illinois University School of Medicine, United States of America

## Abstract

Routine use of antibiotics at subtherapeutic levels in animal feed drives the emergence of antimicrobial resistance. Development of antibiotic-alternative approaches to disease control and prevention for food animals is imperatively needed. Previously, we showed that butyrate, a major species of short-chain fatty acids (SCFAs) fermented from undigested fiber by intestinal microflora, is a potent inducer of endogenous antimicrobial host defense peptide (HDP) genes in the chicken (PLoS One 2011, 6: e27225). In the present study, we further revealed that, in chicken HD11 macrophages and primary monocytes, induction of HDPs is largely in an inverse correlation with the aliphatic hydrocarbon chain length of free fatty acids, with SCFAs being the most potent, medium-chain fatty acids moderate and long-chain fatty acids marginal. Additionally, three SCFAs, namely acetate, propionate, and butyrate, exerted a strong synergy in augmenting HDP gene expression in chicken cells. Consistently, supplementation of chickens with a combination of three SCFAs in water resulted in a further reduction of *Salmonella enteritidis* in the cecum as compared to feeding of individual SCFAs. More importantly, free fatty acids enhanced HDP gene expression without triggering proinflammatory interleukin-1β production. Taken together, oral supplementation of SCFAs is capable of boosting host immunity and disease resistance, with potential for infectious disease control and prevention in animal agriculture without relying on antibiotics.

## Introduction

Widespread use of antibiotics as growth promoters in animal feed is suspected to be a major driving force for the development of antibiotic-resistant pathogens, which have become a critical public health concern worldwide. Enhancing host immunity and disease resistance by specifically boosting the synthesis of endogenous host defense peptides (HDPs) may represent a promising antibiotic-alternative strategy. HDPs have been found in nearly all forms of life and play an important role in the first line of defense [1]–[3]. HDPs kill a broad range of microbes including bacteria, fungi, parasites, and enveloped viruses mainly through physical interaction and disruption of the membranes [1]–[3]. It is, therefore, extremely difficult for pathogens to develop resistance [1]–[3]. In addition to their direct antimicrobial activities, HDPs play a profound role in potentiating the immune response to infections by recruiting and activating immune cells, binding and neutralizing bacterial endotoxins, and promoting wound healing [1]–[4]. Because of these pleiotropic effects, it is beneficial to specifically enhance the synthesis of endogenous HDPs for disease control and prevention.

As an important source of energy and constituents of cellular membranes, fatty acids are represented by a large group of carboxylic acids with an aliphatic hydrocarbon chain that are either saturated or unsaturated. Based on the number of carbon atoms in the aliphatic chain, fatty acids are broadly classified into three groups, namely short-chain fatty acids (SCFAs) (≤ C5), medium-chain fatty acids (MCFAs) (C6 to C11), and long-chain fatty acids (LCFAs) (≥ C12) [5]. Free fatty acids are known to have direct antibacterial activities [6]. Although it remains elusive how fatty acids exert their antibacterial effects, the main mechanism appears to target the bacterial cell membrane by disrupting membrane structure, electron transport, proton gradient or membrane potential [7]. Additionally, MCFAs and SCFAs, except for formic and acetic acids, were found to reduce the invasion and colonization of *Salmonella* to intestinal epithelial cells through suppression of multiple genes required for invasion [8], [9]. Because of their antibacterial capacity, several fatty acids are being used as antimicrobials in human medicine and animal agriculture and as preservatives in food industry [7].

In addition to acting directly on the pathogens, fatty acids were recently found to contribute to disease resistance by acting on the host through induction of HDP gene expression. SCFAs including butyrate and propionate are capable of inducing LL-37 synthesis [10] and LCFAs including lauric acid, palmitic acid, and oleic acid are strong inducers of β-defensin-2 in human cells [11]. The HDP-inducing activity of butyrate was found to be largely due to the ability to inhibit histone deacetylases (HDACs) [12]–[14], which is known to promote hyper-acetylation of the lysine residues in nucleosome core histones, leading to a less compact chromatin and transcriptional activation of a subset of genes [15], [16]. Consistently, several other histone deacetylase inhibitors are also capable of inducing HDP gene expression in humans, albeit with varying potencies [12], [17].

We recently found that butyrate enhances HDP expression in chickens [18]. In the present study, we further compared the relative potency in HDP induction among free fatty acids of various aliphatic chain lengths (C1 to C18). We showed that the HDP expression is regulated inversely with the length of hydrocarbon chain, with SCFAs being the strongest inducers. The presence of double bonds in the aliphatic tails of fatty acids appeared to potentiate HDP induction. We further revealed a strong synergy among three SCFAs including acetate, propionate, and butyrate in enhancing HDP expression and reducing bacterial colonization in the chicken, suggesting the potential for dietary supplementation of SCFAs individually or in combination in disease control and prevention.

## Materials and Methods

### Ethics Statement

This study was carried out in strict accordance with the recommendations in the Guide for the Care and Use of Laboratory Animals of the National Research Council. All animal procedures reported herein were approved by the Institutional Animal Care and Use Committee of Oklahoma State University under protocol no. AG0610. Prior to sample collection, chickens were euthanized by an intramuscular injection of a cocktail of ketamine/xylazine, followed by cervical dislocation to minimize pain.

### Chemicals

Sodium formate (C1), acetate (C2), propionate (C3), butyrate (C4), valeric acid (C5), hexanoate/caproate (C6), *n*-octanoate/caprylate (C8), decanoate/caprate (C10), linoleic acid [C18:2(*n*-6)], α-linolenic acid [C18:3(*n*-3)], and conjugated linoleic acid (CLA) and trichostatin A (TSA) were purchased from Sigma-Aldrich (St. Louis, MO), whereas sodium heptanoate/enanthate (C7), nonanoate/pelargonate (C9), dodecanoate/laurate (C12), tetradecanoate/myristate (C14), octadecanoate/stearate (C18), oleate [C18:1(*n*-9)] were from TCI America (Portland, OR). All free fatty acids were purchased in the sodium salt form, except for valeric acid, linoleic acid, linolenic acid, and CLA, which are in the free acid form. SCFAs (sodium formate, acetate, propionate, butyrate, and valeric acid) and MCFAs (hexanoate, heptanoate, *n*-octanoate, nonanoate, and decanoate) were dissolved in RPMI 1640 medium, while LCFAs (dodecanoate, tetradecanoate, octadecanoate, and oleate) were dissolved in methanol and linoleic acid, α-linolenic acid and CLA were dissolved in ethanol. Bacterial lipopolysaccharide (LPS) from *E. coli* O111:B4 was purchased from Sigma-Aldrich and dissolved in RPMI 160 medium.

### Isolation, Culture, and Stimulation of Chicken Cells

Chicken HD11 macrophages [19] (kindly provided by Dr. Hyun S. Lillehoj from the USDA-ARS) were cultured in 6-well plates in RPMI 1640 containing 10% fetal bovine serum (FBS) and 1% streptomycin/penicillin at 2×10^6^ cells/well. After overnight growth, HD11 cells were incubated with various fatty acids. Chicken peripheral blood mononuclear cells (PBMCs) were isolated from EDTA-anticoagulated venous blood by gradient centrifugation using Histopaque 1077 (Sigma). Cells in the interphase were collected, washed with Hank’s balanced salt solution (HBSS), and then resuspended in RPMI 1640 containing 10% FBS, 1% streptomycin/pencillin, and 20 mM HEPES in 60-mm tissue culture dishes at 6×10^7^ cells/dish. After overnight incubation at 37°C and 5% CO_2_, non-adherent cells were washed off with HBSS, and adherent monocytes were used subsequently for stimulation with fatty acids. Each treatment was performed in duplicate, and all experiments were repeated at least 2–3 times. For each experiment, an equal amount of solvents was added to cells as negative control, and none of the solvents was found to have any appreciable effect on HDP gene expression.

### Analysis of Chicken Gene Expression by Real Time RT-PCR

Following stimulation, cells were harvested in RNAzol RT (Molecular Research Center, Cincinnati, OH), and total RNA was extracted according to the manufacturer’s instructions. The first-strand cDNA was synthesized from 300 ng of total RNA with QuantiTect Reverse Transcription Kit (Qiagen), and real-time PCR was performed with QuantiTect SYBR Green PCR Kit (Qiagen) using 1/40 (for GAPDH) or 1/10 (for HDPs) of the first-strand cDNA and gene-specific primers in a total volume of 10 µl as previously described [18], [20]–[22]. The PCR was set for initial denaturation at 95°C for 10 min, followed by 40 cycles of 94°C for 15 sec, 55°C for 20 sec, and 72°C for 30 sec. Melt curve analysis was performed to ensure the specificity of PCR amplification. Chicken glyceraldehyde-3-phosphate dehydrogenase (GAPDH) was used as a reference for data normalization. The forward and reverse primers for chicken GAPDH, HDPs (AvBD9 and cathelicidin B1), and proinflammatory cytokines (IL-1β, IL-8, and IL-12p40) were previously described [18]. Relative changes in the gene expression level were calculated using the ΔΔCt method as described [18], [20]–[22].

### Histone Deacetylase (HDAC) Activity Assay

The HDAC activity assay was performed using the Fluor-de-Lys® HDAC Fluorimetric Cellular Activity Assay Kit (Enzo Life Sciences) according to the manufacturer’s instructions. Chicken HD11 cells (1×10^5^) were cultured in phenol red-free RPMI 1640 containing 10% FBS in a 96-well tissue culture plate overnight. Cells were treated in duplicate with or without SCFAs in the presence of 100 µM of Fluor-de-Lys®, a fluorogenic, cell-permeable HDAC substrate for 4 h. The deacetylation reaction was then stopped by addition of TSA, a strong HDAC inhibitor, in a cell lysis buffer containing 1% NP-40. The fluorescent signal was generated by addition of a developer solution to a final concentration of 1 µM, and the fluorescence was recorded at 360 nm excitation and 460 nm emission using FLx800 Multi-Detection Microplate Reader (Bio-Tek Instruments). The HDAC inhibitory activity (%) was calculated as [1–(*F*_treatment_–*F*_background_)/(*F*_max_–*F*_background_)]×100, where *F*_treatment_ is the fluorescence of cells exposed to SCFAs, *F*_max_ is the maximum fluorescence of cells without being exposed to SCFAs, and *F*_background_ is the fluorescence of cell culture medium without cells.

### Oral Supplementation of SCFAs and Experimental Infection of Chickens with *Salmonella enteritidis*

A total of 20, day-of-hatch male Cornish Rock broiler chickens were purchased from a commercial hatchery (Ideal Poultry, Cameron, TX) and randomly divided into four groups of 5 birds with free access to a standard antibiotic-free ration and deionized water for 4 days. Water containing 0.5% sodium acetate, 0.2% propionate and/or 0.1% butyrate was provided ad libitum for each group for the next 2 days, prior to an intraesophageal infection with 0.5 ml of Lysogeny broth (LB) containing 1×10^7^ colony forming units (CFU) of *Salmonella enteritidis* phage type 13a (a kind gift from Dr. Susan Lamont at Iowa State University) [23]. SCFAs were administered in water for another 4 days, before the birds were euthanized and cecal contents were aseptically collected from each animal, weighed, serially diluted in PBS, and plated on Brilliant Green agar plates (Becton Dickinson) containing 20 µg/ml of nalidixic acid (Sigma-Aldrich) for overnight growth and bacterial enumeration. The animal trial was carried out under strict ABSL-2 conditions, and all procedures were approved.

### Statistical Analysis

Unpaired Student’s two-tailed t-test was used to evaluate the statistical significance between treatments using GraphPad Prism 5 (GraphPad Software, La Jolla, CA). P<0.05 was considered statistically significant.

## Results

### Inverse Correlation between the HDP-inducing Activity and the Aliphatic Chain Length of Free Fatty Acids

To first test the cytotoxicity of free fatty acids, we incubated chicken macrophage HD11 cells and primary monocytes with different fatty acids in a broad range of concentrations for 24 h and then examined their toxicity to chicken HD11 cells and primary monocytes using alamarBlue as described [24], [25]. Only subtoxic concentrations were then used to examine the relationship between the aliphatic hydrocarbon chain length of each fatty acid and its HDP-inducing capacity. Different concentration ranges were used in many cases in order to show the magnitude of the peak response for each fatty acid. Chicken β-defensin 9 (*AvBD9*) and *cathelicdin B1*, which are readily induced by sodium butyrate [18], were used as representative members of the defensin and cathelicidin gene families, and gene expression changes were evaluated by real-time RT-PCR.

**Figure 1:**
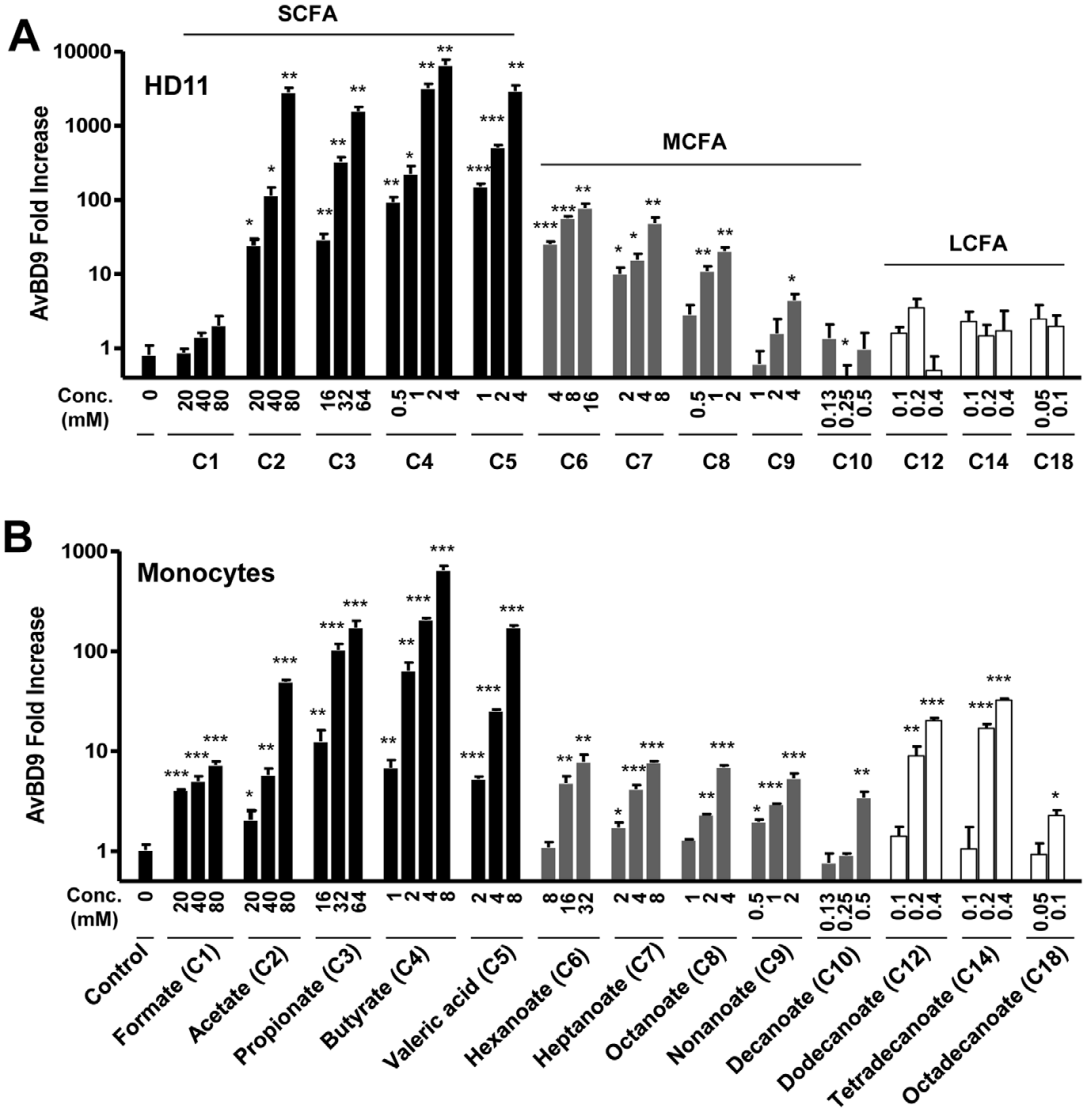
Regulation of *AvBD9* gene expression by free fatty acids. Chicken macrophage HD11 cells (A) and primary monocytes (B) were treated in duplicate with or without indicated concentrations of short-chain fatty acids (SCFA), medium-chain fatty acids (MCFA) or long-chain fatty acids (LCFA) for 24 h, followed by real-time RT-PCR analysis of *AvBD9* gene expression. Data was normalized with GAPDH, and relative fold change of each treatment versus solvent control was calculated using ΔΔCt method. Data shown are means ± standard error of a representative of 2–3 independent experiments. It is noted that all fatty acids were used at subtoxic concentrations and, because of different toxicities to HD11 cells and primary monocytes, slightly different concentrations of free fatty acids were used in the two cell types in a few cases in order to show the optimal AvBD9-inducing activity in each cell type. *P<0.05, **P<0.01, and ***P<0.001 (in comparison with solvent controls by unpaired Student’s *t*-test).

As shown in Fig. 1A, we observed a clear dose-dependent induction of the *AvBD9* mRNA in HD11 cells in response to individual SCFAs and MCFAs, with LCFAs being largely inactive. A peak response occurred with SCFAs, with greater than 1000-fold induction of the *AvBD9* gene expression in HD11 cells when exposed to 80, 64, 4, and 4 mM of sodium acetate, propionate, butyrate, and valeric acid respectively. The magnitude of the *AvBD9* induction was dramatically reduced with MCFAs, with a maximal increase of less than 100-fold seen in HD11 cells (Fig. 1A). A similar trend was also observed in primary chicken monocytes, with SCFAs being the most potent inducers (Fig. 1B). Among SCFAs, butyrate has the strongest capacity to induce *AvBD9* gene expression, followed by valeric acid, sodium propionate, and acetate, with sodium formate being minimally active in both HD11 cells and primary monocytes. However, a notable cell-specific regulation of *AvBD9* expression was observed. Being largely inactive in HD11 cells (Fig. 1A), LCFAs including dodecanoate/laurate (C12), tetradecanoate/myristate (C14), and octadecanoate/stearate (C18) maintained a comparable, if not slightly better, *AvBD9*-inducing activity than MCFAs in primary monocytes (Fig. 1B).

**Figure 2:**
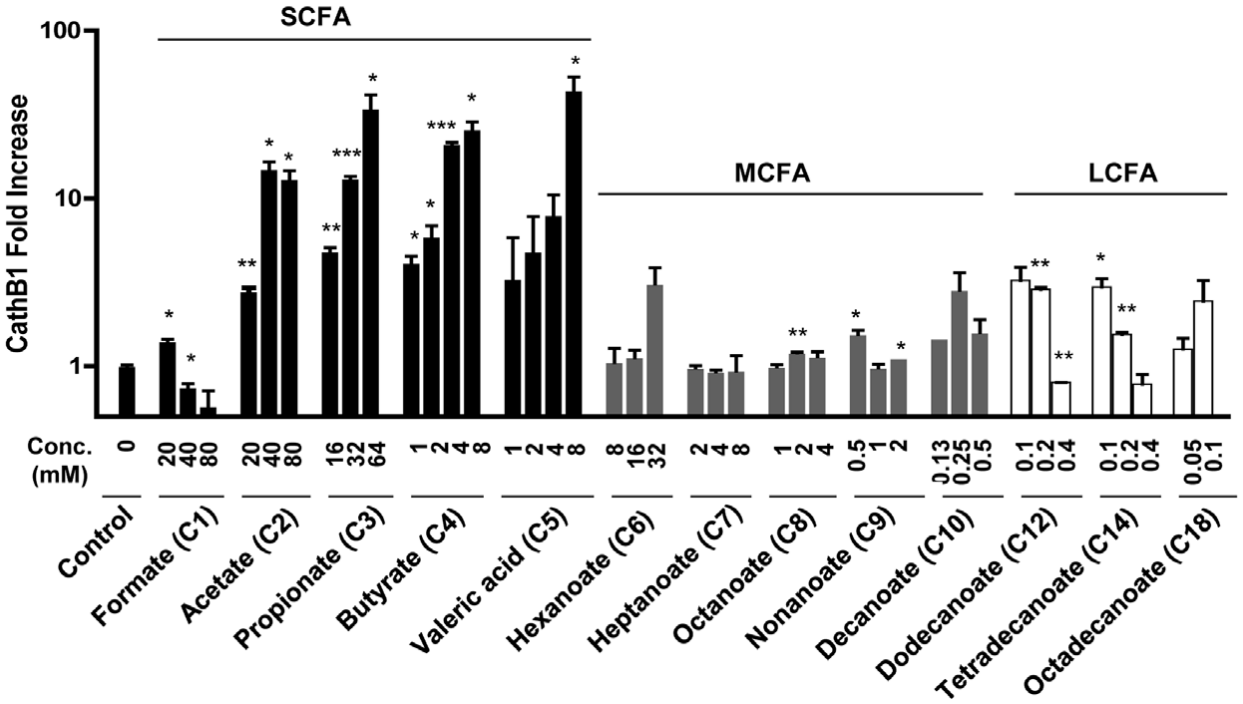
Modulation of cathelicidin B1 gene expression by free fatty acids. Primary chicken monocytes were treated in duplicate with or without indicated concentrations of short-chain fatty acids (SCFA), medium-chain fatty acids (MCFA) or long-chain fatty acids (LCFA) for 24 h, followed by real-time RT-PCR analysis of *cathelicidin B1* gene expression. Data was normalized with *GAPDH*, and relative fold change of each treatment versus solvent control was calculated using ΔΔCt method. Data shown are means ± standard error of a representative of 2–3 independent experiments. *P<0.05, **P<0.01, and ***P<0.001 (in comparison with solvent controls by unpaired Student’s *t*-test).

Similar to *AvBD9*, *cathelicidin B1* was readily induced by SCFAs including sodium acetate, propionate, butyrate, and valeric acid in both HD11 cells (data not shown) and primary monocytes, with sodium formate being mostly inactive (Fig. 2). The maximum increases in the *cathelicidin B1* expression in monocytes were between 20- to 40-fold among C2–C5 SCFAs. However, in contrast to *AvBD9*, *cathelicidin B1* was barely induced by any of the MCFAs and LCFAs in either HD11 cells (data not shown) or monocytes (Fig. 2), suggestive of differential regulation of HDPs by fatty acids. It is worth noting that, all fatty acids tested above involved the use of the sodium salt form, with exception of valeric acid, because of an inability to find a commercial source of a salt form. Nevertheless, we do not expect much difference in the HDP-inducing activity between the acid and salt form of SCFAs, as we only observed a minimal, less than 2-fold difference in the *AvBD9* induction in HD11 cells between propionic and butyric acids and their respective sodium salt forms (data not shown).

**Figure 3:**
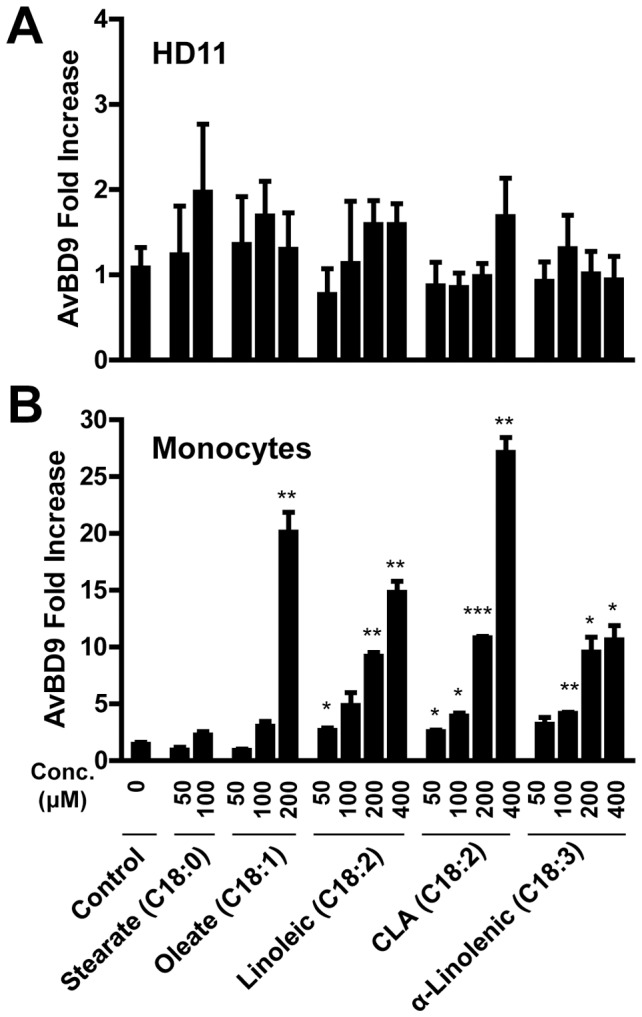
Differential expression of *AvBD9* in response to unsaturated fatty acids. Chicken HD11 macrophage cells (A) and primary monocytes (B) were treated in duplicate with different concentrations of sodium stearate, sodium oleate, linoleic acid, conjugated linolenic acid (CLA), and α-linolenic acid for 24 h, followed by real-time RT-PCR analysis of *AvBD9* gene expression. Data shown are means ± standard error of a representative of 2–3 independent experiments. Because of an obvious cytotoxicity, 200 and/or 400 µM could not be tested for sodium stearate and oleate. *P<0.05, **P<0.01, and ***P<0.001 (in comparison with solvent controls by unpaired Student’s *t*-test).

To further examine the effect of the saturation status of hydrocarbon chain on HDP expression, different concentrations of saturated C18 fatty acid (sodium stearate/octadecanoate) as well as unsaturated C18 fatty acids including sodium oleate [C18:1(n-9)], linoleic acid [C18:2(*n*-6)], CLA (C18:2), and α-linolenic acid [C18:3(*n*-3)] were used to stimulate HD11 cells for 24 h. Real-time RT-PCR revealed that, similar to saturated stearate, all tested unsaturated LCFAs failed to induce the *AvBD9* gene expression in HD11 cells (Fig. 3A), but showed an obvious dose-dependent *AvBD9* induction in chicken primary monocytes (Fig. 3B). It is interesting to note that all unsaturated C18 fatty acids appear more potent in enhancing the *AvBD9* gene expression than saturated stearate in chicken monocytes. Overall, these findings surprisingly suggested the involvement of double bonds in the regulation of HDP expression. However, no direct correlation between the saturation status and HDP-inducing activity was observed, as all unsaturated C18 fatty acids showed a comparable ability to activate HDP gene transcription regardless of the number of double bonds.

### Impact of Free Fatty Acids on the Inflammatory Response in HD11 Cells

**Figure 4:**
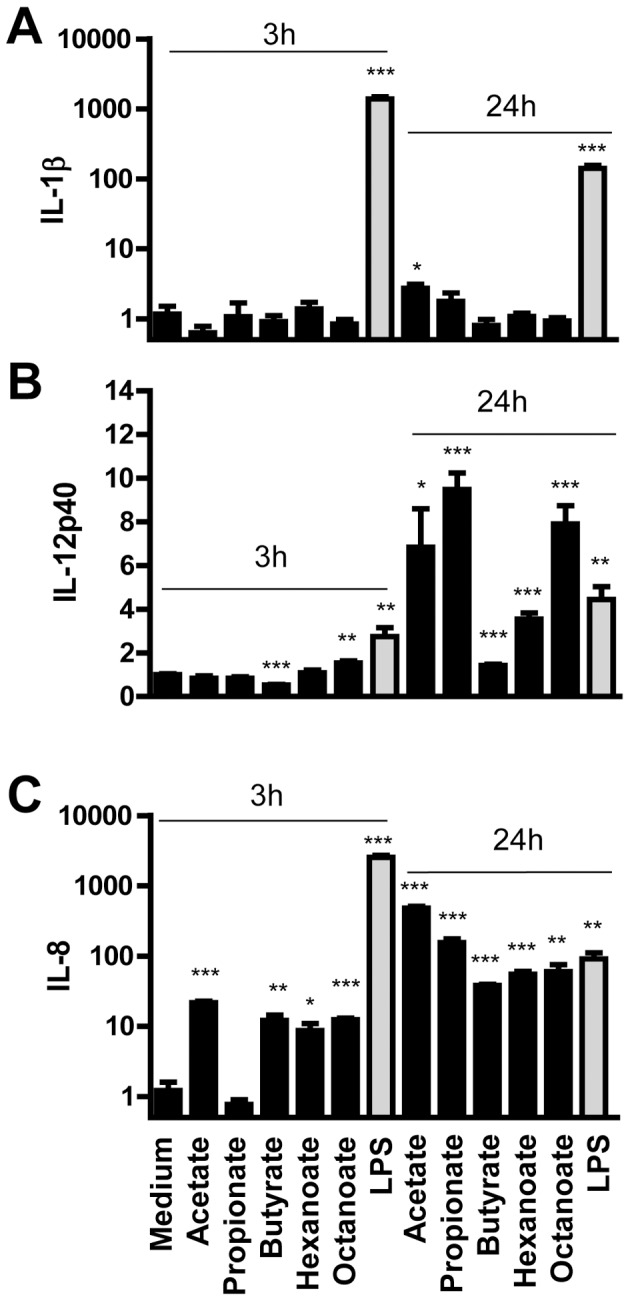
A minimum impact of free fatty acids on the expression of proinflammatory cytokines. Chicken HD11 cells were stimulated with different fatty acids at optimal HDP-inducing concentrations (80 mM acetate, 32 mM propionate, 4 mM butyrate, 16 mM hexanoate, and 2 mM octanoate) or LPS (1 µg/ml) as a positive control for 3 and 24 h, followed by real-time RT-PCR analysis of the expression of *IL-1β* (A), *IL-12p40* (B), and *IL-8* (C). Data shown are means ± standard errors from 2–3 independent experiments. *P<0.05, **P<0.01, and ***P<0.001 (in comparison with solvent controls by unpaired Student’s *t*-test).

SCFAs, particularly butyrate, generally exert anti-inflammatory effects and have been used to treat inflammatory bowel diseases [26], [27]. To confirm augmentation of HDP gene expression by free fatty acids without triggering a proinflammatory response, we treated HD11 cells with or without different fatty acids at optimal HDP-inducing concentrations for 3 and 24 h and analyzed the expressions of three representative cytokines including *IL-1β, IL-8*, and *IL-12p40*. Bacterial lipopolysaccharide (LPS) from *E. coli* O111:B4 at 1 µg/ml was used as a positive control. All representative fatty acids, including acetate, propionate, butyrate, hexanoate, and octanoate, had essentially no effect on *IL-1β* at both time points (Fig. 4). No influence on *IL-12p40* expression was observed following fatty acid stimulation for 3 h; however, a 3- to 10-fold induction was seen with all fatty acids except for butyrate. As compared with LPS that caused>1000-fold induction, a minimum influence (∼10-fold increase) on *IL-8* expression was observed with all tested fatty acids except for propionate after 3 h stimulation (Fig. 4). All fatty acids showed an *IL-8*-inducing activity comparable to LPS after 24 h (Fig. 4). Taken together, these results demonstrated that fatty acids generally have no or a limited influence on triggering the inflammatory response while promoting HDP synthesis.

### Synergistic Induction of *AvBD9* Expression and Reduction of Bacterial Colonization by SCFAs

**Figure 5:**
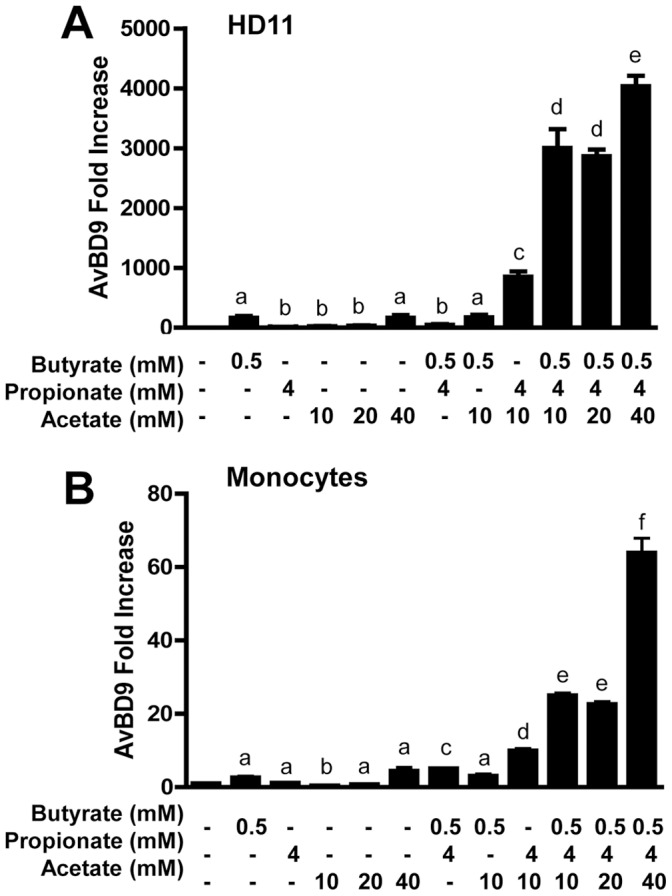
Synergistic induction of *AvBD9* with acetate, propionate, and butyrate in chicken HD11 cells (A) and primary monocytes (B). Cells were incubated with acetate, propionate, and butyrate alone or in combinations for 24 h, followed by real-time RT-PCR analysis of *AvBD9* expression. Data shown are means ± standard errors from 3 independent experiments. The bars without common superscript letters denote significance (*P*<0.05 by unpaired Student’s *t*-test).

Because acetate, propionate, and butyrate are among the most potent fatty acids in inducing *AvBD9* gene expression and they also represent the major species of SCFAs being produced simultaneously by intestinal microflora, we sought to determine the synergistic effect of these three SCFAs on HDP synthesis. Chicken HD11 cells and primary monocytes were treated with acetate, propionate, and butyrate individually or in combinations for 24 h and followed by real-time RT-PCR analysis of *AvBD9* gene expression. Individual SCFAs at low concentrations gave a minimum induction of *AvBD9* gene. A combination of propionate and acetate showed an obvious synergism in both HD11 cells (Fig. 5A) and primary monocytes (Fig. 5B). However, no synergy was observed with combined use of butyrate/propionate or butyrate/acetate. Strikingly, an addition of all three SCFAs resulted in a 25- to 50-fold further induction of the *AvBD9* gene in both cell types when compared to individual fatty acids (Fig. 5). Nevertheless, no obvious synergism in *cathelicidin B1* gene expression was observed with any combination of two or three SCFAs in either cell type (data not shown), suggesting that *cathelicidin B1*and *AvBD9* are differentially regulated.

**Figure 6:**
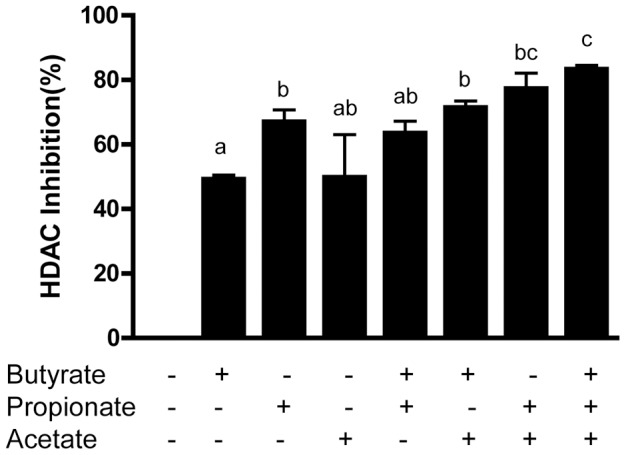
Inhibition of the HDAC activity by acetate, propionate, and butyrate. Chicken HD11 cells were incubated in duplicate with or without three SCFAs in the presence of Fluor-de-Lys®, a fluorogenic, cell-permeable HDAC substrate for 4 h. The deacetylation reaction was stopped and the fluorescent signal was generated by addition of a developer solution containing trichostatin A and NP-40. Fluorescence was monitored at 360 nm excitation and 460 nm emission. HDAC inhibition by SCFAs was calculated relative to the cells without being exposed to any HDAC inhibitor. Data shown are means ± standard errors. The bars without common superscript letters denote significance (*P*<0.05 by unpaired Student’s *t*-test).

SCFAs and butyrate in particular are well-known histone deacetylase inhibitors [13], [14]. To study the impact of histone deacetylation on the *AvBD9*-inducing activity in chickens by SCFAs, we treated HD11 cells with or without acetate, propionate, and butyrate individually or in combination for 4 h and then performed HDAC assays using Fluor-de-Lys® HDAC Fluorimetric Cellular Activity Assay Kit (Enzo Life Sciences). As shown in Fig. 6, low concentrations of butyrate (0.5 mM) and acetate (40 mM) showed a similar HDAC inhibitory activity of approximately 50%, while propionate (4 mM) suppressed the HDAC activity by 67% (Fig. 6). Moreover, a combination of two SCFAs showed either comparable or higher HDAC inhibitory activity than any individual SCFA. More importantly, a simultaneous treatment of HD11 cells with all three SCFAs resulted in the greatest inhibition of the HDAC activity (83%) (Fig. 6). Consistent with the *AvBD9*-inducing activity, a combination of propionate/acetate showed a higher HDAC inhibition than any other combination of two SCFAs. These results are correlated well with the relative capacity of SCFAs to stimulate *AvBD9* gene expression (Fig. 5).

**Figure 7:**
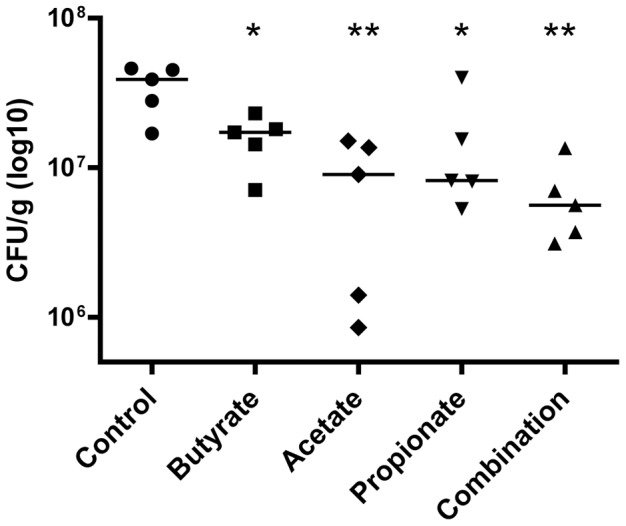
Synergistic reduction of the *Salmonella enteritidis* load in the cecum of chickens by a combination of acetate, propionate and butyrate. Four day-old male broiler chicks were supplemented with or without 0.5% acetate, 0.2% propionate, and 0.1% butyrate alone or in combinations in water for 2 days with 5 birds per group, followed by an inoculation with *S. enteritidis* phage type 13a (1×10^7^). SCFA supplementation was continued for another 4 days before the cecal content was collected and bacterial number enumerated. Each dot indicates the bacterial titer in a bird and the solid line represents the median value of each treatment. *P<0.05 and **P<0.01 (by unpaired Student’s *t*-test).

To further confirm whether SCFA-mediated synergistic induction of *AvBD9* could confer animals an enhanced resistance to bacterial infection, we fed 4-day-old male broiler chickens with 0.5% acetate, 0.2% propionate, and 0.1% butyrate individually or in combination in water for 2 days, followed by an inoculation with 1×10^7^ CFU of *S. enteritidis* phage type 13a for another 4 days. The bacterial titer in the cecal content was examined. As seen in Fig. 7, a significant reduction of the *S. enteritidis* titer was observed with supplementation of acetate, propionate, and butyrate individually. Importantly, the most dramatic reduction (∼7-fold) in bacterial colonization was seen in the chickens receiving a combination of three SCFAs, consistent with their ability to induce *AvBD9* gene expression in vitro (Fig. 5) and that AvBD9 (formerly known as gallinacin-6) is a broad-spectrum antimicrobial expressed throughout the chicken gastrointestinal tract with the capacity to kill multiple species of enteric pathogens including *Salmonella* [28].

## Discussion

It is well-known that free fatty acids possess a direct antibacterial activity [6], [7]. Among all saturated fatty acids, MCFAs generally have the highest antibacterial activity peaking with a chain length around 10–12 carbons, and such an activity tends to decrease as the hydrocarbon chain gets longer or shorter [6], [7]. In the present study, we compared for the first time the relative HDP-inducing potency of free fatty acids ranging from 1 to 18 carbons. In contrast to their antibacterial activity, SCFAs, but not MCFAs or LCFAs, have the strongest capacity to induce the expression of HDP genes in chicken HD11 cells and primary monocytes, with butyrate, a 4-carbon fatty acid being the most potent. However, although LL-37 is minimally induced, the expressions of β-defensin 2 and, to a lesser extent, β-defensin 3 are markedly increased in human sebocytes in response to LCFAs (lauric acid, palmitic acid, and oleic acid) [11], suggesting that LCFAs may have the capacity to target a subset of HDP genes in humans, which is yet to be confirmed in chickens and other animal species.

Saturation status of free fatty acids is known to affect the antibacterial activity. Unsaturated fatty acids tend to be more potent than saturated fatty acids with the same hydrocarbon chain length [7]. The number of double bonds is generally positively correlated with the antibacterial activity of unsaturated fatty acids. In contrast with saturated free fatty acids, the most antibacterially active monounsaturated fatty acids usually consist of 14–16 carbons [7]. We now show that the presence of double bonds also seems to potentiate the HDP-inducing activity of free fatty acids, which coincides with many health benefits associated with unsaturated fatty acids. However, based on our limited results with oleate [C18:1(*n*-9)], linoleic acid [C18:2(*n*-6)], CLA (C18:2), and α-linolenic acid [C18:3(*n*-3)], we failed to observe a clear correlation between the abundance of double bonds of fatty acids and the HDP-inducing activity. Apparently, testing additional unsaturated fatty acids of different hydrocarbon chain lengths for their HDP-inducing activity and studying the underlying mechanisms warrant further investigations.

Acetate, propionate, and butyrate are the major species of SCFAs produced simultaneously by bacterial fermentation of undigestable carbohydrates in the intestine [26], [27], [29]. The concentrations of acetate, propionate, and butyrate are averaged 54.0, 11.6, and 11.1 µmol/g, respectively, in adult human fecal samples [30] and 33.2, 12.0, and 5.8 µmol/g, respectively, in the cecal content of 18-day-old chickens [8]. To our surprise, we observed a strong synergy in *AvBD9* gene induction in response to a combination of three major SCFAs. Furthermore, an oral supplementation of three SCFAs, based roughly on their in vivo molar ratio, led to a reduction in cecal *Salmonella* in a synergistic manner, suggestive of physiological benefits of simultaneous natural production of multiple SCFAs in the lower gut. Importantly, such a synergy was observed most dramatically when suboptimal HDP-inducing concentrations of SCFAs are used. Given the rapid metabolism and absorption in the upper gut [31], oral supplementation of SCFAs is predicted to result in only a small fraction reaching the lower gut where most foodborne pathogens colonize. Therefore, it is hugely beneficial to supplement multiple SCFAs simultaneously in the diets for control of infections.

It is worth mentioning that, the antibacterial concentrations of SCFAs and MCFAs were generally in 10–100 mM concentrations [8], [9], whereas the doses to induce peak HDP synthesis are 2–8 mM (Fig. 1). In fact, we observed no inhibitory effect of sodium butyrate, propionate, and acetate against *S. enteritidis* phage type 13a in a standard broth microdilution assay [32] when used at 0.1%, 0.2%, and 0.5%, respectively, either individually or in combinations (data not shown). These are also the concentrations used in vivo in the animal trial when a decline in the cecal bacterial load was observed (Fig. 7). We also showed previously that sodium butyrate enhanced the antibacterial activity of chicken monocytes through induction of HDP synthesis, but not due to a change in phagocytosis, oxidative burst or activation status of the phagocytes [18]. Although MCFAs and SCFAs are individually capable of reducing bacterial invasion and colonization to intestinal epithelial cells at the doses slightly lower than the antibacterial concentrations, a combination of acetate, propionate, and butyrate mimicking the in vivo concentrations showed no impact on bacterial invasion [8], [9], suggesting the influence of SCFAs on bacterial invasion may not have much in vivo relevance. Given quick metabolism of free fatty acids, the observation that oral supplementation of SCFAs [33] or MCFAs [9] caused a reduction of *Salmonella* colonization in the cecum of chickens is likely due to the HDP-inducing rather than direct antibacterial or bacterial invasion-inhibitory activity of fatty acids.

SCFAs are well-known HDAC inhibitors and were recently found to induce HDP synthesis mainly through inhibition of HDACs in humans [12]. Among SCFAs, butyrate is the most potent HDAC inhibitor, with valerate and propionate being moderate and acetate least effective [34]. Here we found that butyrate has the highest efficacy in HDP-induction, followed by valerate, propionate, and acetate (Figs. 1 and 2), showing a strong correlation with their ability to suppress HDACs. Consistently, as compared to individual SCFAs, a combination of three exhibits the strongest capacity to induce HDPs (Fig. 5) and reduces bacterial colonization (Fig. 7), while showing the highest HDAC inhibitory activity (Fig. 6). It will be important to screen other known HDAC inhibitors for their ability to induce HDP production and enhance disease resistance.

In summary, we revealed for the first time that, in comparison with MCFAs and LCFAs, SCFAs are the most potent HDP inducers. We also found that a combination of SCFAs, particularly at low concentrations, leads to a synergistic induction of HDPs, which has important practical implications in animal production practice. Because of a plethora of beneficial effects on bacterial suppression and host immune augmentation with a minimum impact on the inflammatory response, free fatty acids and SCFAs in particular have strong potential for disease control and prevention and may represent promising alternatives to antibiotics. Given that fatty acids also induce HDP synthesis in humans, SCFA-mediated potentiation of host immunity is expected to be broadly applicable to all major animal species including humans.
